# Automatic Optical Path Alignment Method for Optical Biological Microscope

**DOI:** 10.3390/s25010102

**Published:** 2024-12-27

**Authors:** Guojin Peng, Zhenming Yu, Xinjian Zhou, Guangyao Pang, Kuikui Wang

**Affiliations:** 1 Key Laboratory of Cognitive Radio and Information Processing, Ministry of Education, Guilin University of Electronic Technology, Guilin 541004, China; pengguojin@mails.guet.edu.cn; 2 Guangxi Key Laboratory of Machine Vision and Intelligent Control, Wuzhou University, Wuzhou 543000, China; wzxykjl1308@163.com (X.Z.); pangguangyao@snnu.edu.cn (G.P.); wk5581798@outlook.com (K.W.)

**Keywords:** biological microscope, optical path alignment, objective identification, weighted circle fitting, alignment evaluation

## Abstract

A high-quality optical path alignment is essential for achieving superior image quality in optical biological microscope (OBM) systems. The traditional automatic alignment methods for OBMs rely heavily on complex masker-detection techniques. This paper introduces an innovative, image-sensor-based optical path alignment approach designed for low-power objective (specifically 4×) automatic OBMs. The proposed method encompasses reference objective (RO) identification and alignment processes. For identification, a model depicting spot movement with objective rotation near the optical axis is developed, elucidating the influence of optical path parameters on spot characteristics. This insight leads to the proposal of an RO identification method utilizing an edge gradient and edge position probability. In the alignment phase, a symmetry-based weight distribution scheme for concentric arcs is introduced. A significant observation is that the received energy stabilizes with improved alignment precision, prompting the design of an advanced alignment evaluation method that surpasses conventional energy-based assessments. The experimental results confirm that the proposed RO identification method can effectively differentiate between 4× and 10× objectives across diverse light intensities and exposure levels, with a significant numerical difference of up to 100. The error–radius ratio of the weighted circular fitting method is maintained below 1.16%, and the fine alignment stage’s evaluation curve is notably sharper. Moreover, tests under various imaging conditions in artificially saturated environments indicate that the alignment estimation method, predicated on critical saturation positions, achieves an average error of 0.875 pixels.

## 1. Introduction

OBMs, indispensable tools in life science research and medical diagnosis, are undergoing a revolutionary upgrade toward intelligence, driven by artificial intelligence technology. With the continuous advancements in optics, electronics, and automation, modern biological microscopes not only have higher resolution and more powerful imaging capabilities but have also achieved intelligent operation and significant expansion of their application fields through the integration of advanced algorithms. These microscopes incorporate advanced technologies such as fluorescence imaging [1], confocal scanning [2], and super-resolution imaging [3], enabling the unprecedented high-precision and high-definition observation of biological samples.

**Figure 1:**
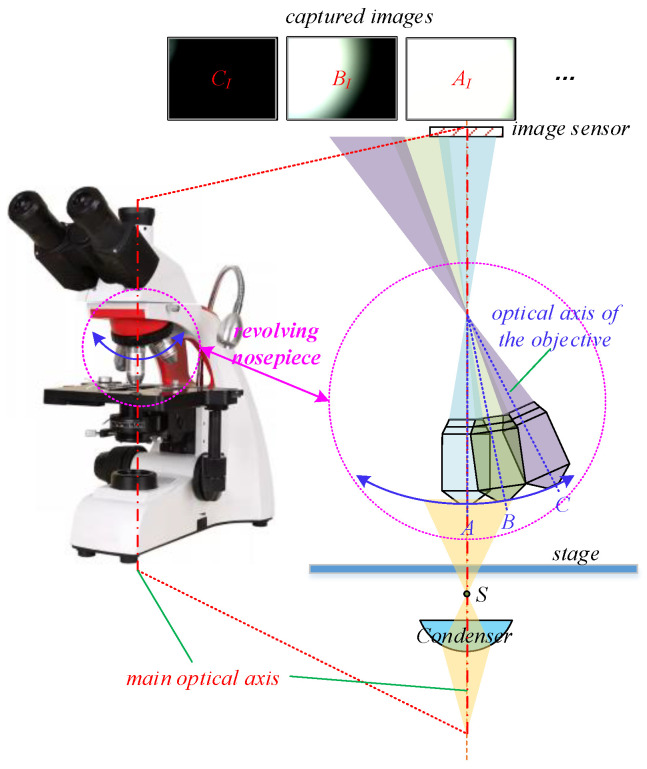
Schematic of the optical path alignment of the objectives. At position *A*, the optical axis of the objective is perfectly aligned with the main optical axis, with the optical path depicted by a pale blue light beam, and the corresponding spot image labeled by $A_{I}$. Positions *B* and *C* illustrate the objective’s optical axis deviating to varying extents from the main optical axis. The optical paths for these positions are delineated by pale green and pale purple beams, respectively, with the corresponding spot images represented by $B_{I}$ and $C_{I}$.

Securing clear and precise microscopic images remains the fundamental objective of OBMs. The degree of intelligence embedded in this process serves as a pivotal benchmark for assessing the overall intelligence quotient of these instruments. To enhance the level of intelligence of microscopes, numerous scholars have devoted considerable efforts to efficient solutions for automatic focusing [4,5,6]. While the precision of focusing plays a pivotal role in determining the imaging quality of a microscope’s optical path, it is not the only determinant. Other factors such as the properties of the light source, the settings of the aperture diaphragms, the degree of optical path alignment, and the effectiveness of aberration correction also significantly shape the overall quality of the optical path. Among the factors mentioned above, an aligned optical path is a key prerequisite for achieving high-quality imaging. In OBMs, the optical axes of components such as the light source, imaging device, and aperture are typically fixed and aligned with the main optical axis, whereas the optical axis of the objective is adjustable. OBMs are typically equipped with a set of objectives with discontinuous magnifications, all neatly arranged on a revolving nosepiece, as shown in Figure 1. According to standard operating protocols, biological microscopes initially use a low-magnification objective for target positioning and rough focusing, followed by switching to a high-magnification objective for detailed observation. Upon switching the objectives, it is crucial to align the objective’s optical axis with the main optical axis, which corresponds to position A depicted in Figure 1. Deviation of the objective’s optical axis from the main optical axis, as indicated at locations B and C in Figure 1, results in reduced image brightness, distortion, and even imaging interruption, severely impacting image quality. Essentially, the optical path alignment in OBMs can be summarized in two key aspects: 1. identifying objectives with specific magnifications; 2. aligning the optical axis of the selected objective with the main optical axis.

In conventional manual microscopes, the identification and switching of the objectives are performed manually, while the alignment and locking of the optical path are dependent on a mechanical limit slot. The limit slot is ideal for manual microscopes but can result in torque imbalance and motor step loss in electronically controlled microscopes, leading to error accumulation. Abandoning the limit slot and supplementing it with an alignment judgment mechanisms can help maintain motor torque balance. A commonly used countermeasure is to incorporate sensors, such as electromagnetic positioning sensors [7], photoelectric positioning sensors [8], and capacitance sensors [9], to provide alignment marks. In this approach, both the identification of objectives and the alignment of the optical path are achieved through the sensing circuit. Numerous manufacturers have equipped all objectives with sensors, as exemplified by the EOCH series of electric objectives made by Shibuya, Nanjing, China. However, additional sensing circuits inevitably complicate the electrical control structure. In practical microscopic imaging systems, the mechanical and electronic components are intricately linked. A slight modification in the electronic control structure of the revolving nosepiece may initiate a chain of local or global mechanical adjustments, potentially leading to numerous issues that impact the overall system’s reliability. Thus, the challenge of achieving automatic optical alignment in OBMs without the need for limit slots or extra sensing circuits, and with minimal mechanical and electronic complexity, has emerged as a highly significant issue worthy of exploration.

Contrasting with the alignment schemes previously discussed, we employed an image sensor (IS) to facilitate the optical path alignment of specific objectives, thereby bypassing the mechanical and electrical intricacies of the sensing circuit. This strategy was primarily derived from the following considerations: Since imaging characteristics are deeply affected by the degree of the optical path alignment, as well as the magnifications of objectives, the guidance information for optical path alignment and the criteria for objective identification can in turn be extracted by analyzing the characteristics of the captured images. This image-based alignment guidance approach not only simplifies the control structure of OBMs but also enhances the flexibility and convenience of optimizing alignment strategies.

In fact, the majority of current autofocus schemes for optical microscopy systems rely on image features, avoiding an increase in structural complexity. The efficacy of the image-feature-based focus guidance mechanism is attributed to the fact that most microscopic imaging systems can be modeled as a unified single-lens configuration. The uniformity in the blur characteristics of out-of-focus images allows for the application of a universal defocus evaluation metric. Conversely, across various microscope systems, there are significant differences not only in the optical paths, the mechanical and electrical control structures, but also in the alignment criteria themselves. Refs. [10,11] have proposed optical path alignment schemes for coherence-controlled holographic microscopes and laser-scanning fluorescence microscopes, respectively. In both schemes, the alignment criterion is based on the coincidence of microspots from the primary and secondary optical paths. The optical path alignment scheme for the stimulated emission depletion microscopes, described in [12], similarly employs the alignment criterion based on the coincidence of two microspots. In transmission electron microscopes, the alignment criterion commonly relies on the center coordinates of the Laue circle [13,14]. Additionally, both the optical path alignment scheme for vertical lathe proposed in [15] and the scheme for mask-free photolithography proposed in [16] utilize the coordinates of a single microspot as the alignment criterion. However, the optical path alignment in OBMs is contingent upon the rotation of the revolving nosepieces, resulting in a single large spot that encompasses the entire field of view (FOV). Consequently, the aforementioned criterion is clearly inapplicable to the OBM system. Hence, it is essential to craft an optical path alignment scheme that is specifically aligned with the unique imaging and structural features of OBMs.

Regarding OBMs, there is a relative scarcity of studies and cases focusing on optical path alignment based on imaging features. OBMs exhibit significant structural differences in their optical paths and electrical controls compared to the complex microscopes mentioned above. Consequently, it is challenging to directly apply the optical path alignment schemes designed for these complex microscopes to OBMs. In contrast, OBMs have a simpler optical path structure, which is more compatible with the alignment structures and mechanisms used in optical communication systems, particularly within optical imaging communication systems. Both systems utilize a light-emitting diode (LED) or laser diode (LD) as the light source and an image sensor as the receiving device, striving for aligned optical paths to maximize the signal-to-noise ratio (SNR). Optical path alignment in optical communication systems necessitates three-dimensional consideration, whereas in biological microscopes, the alignment is confined to two dimensions due to the path constraints of the revolving nosepiece. Therefore, the optical path alignment mechanisms employed in optical communication systems are more readily adaptable to OBMs. Numerous scholars have delved into the challenges of optical path alignment and tracking within optical communication systems, proposing a variety of solutions. These solutions are primarily categorized into two distinct approaches: those based on the spot feature and those based on the received intensity.

In schemes for optical path alignment that incorporate spot features, small spots are often regarded as perfect points for tracking purposes, as demonstrated in [17,18], which consider near-field laser spots to be point sources. However, when a laser beam undergoes dispersion or distortion, the spot enlarges and deforms, making it unsuitable for approximation as a point. To address this issue, studies [19,20] employed the centroid for aligning the optical path. Zhao H. et al. [21] improved upon this by refining the spot center estimation with a background noise baseline and the OTSU method. Likewise, the center of the received spot acts as a fairly precise indicator of the direction of the optical path. Methods such as circle fitting [22,23] and circle detection [24] are effective for pinpointing the spot’s center. When the radial intensity distribution of the received spot approximates a Gaussian profile, the spot’s center can be estimated through Gaussian function fitting [25]. Additionally, some researchers have employed the linear term of the near-field phase to ascertain the center of the spot within optical systems [26].

The idea behind the received-intensity-based optical path alignment schemes is relatively straightforward. The intensity detected by the receiver increases as the incident angle of light decreases, and their relationship exhibits a unimodal curve [27], with the peak nearly aligning with the position where the optical path is optimally aligned. Accordingly, an evaluation mechanism for optical path alignment is established, and peak search strategies [28,29] are used to gradually correct the optical path. To accelerate the acquisition and alignment of the optical path, studies [25,30], respectively, fit the evaluation curves derived from LED-PD and LD-PD systems with Gaussian functions and applied the extended Kalman filter algorithm for optical path tracking.

In summary, spot-feature-based methods can swiftly ascertain the degree of optical path deviation, yet they are prone to estimation errors due to the non-ideal nature of the optical path. In contrast, the received-intensity-based methods more directly address the fundamental requirement of optical systems—the received SNR—and offer more accurate alignment guidance. Although the step search strategy in the received-intensity-based methods are more time-consuming, they achieve higher accuracy.

The optical path in an OBM is commonly modeled as a single-lens system [31], with its point spread function closely approximating a Gaussian function [32]. In the OBM system depicted in Figure 1, the convergence position of the beam passing through the condenser, denoted by *S*, can be simply treated as an off-focus point source. Therefore, without a slide and with received intensity below saturation, the intensity distribution within the spot formed by *S* closely follows a Gaussian distribution, simplifying the estimation of the spot’s center. The saturation of received intensity invalidates the Gaussian model, yet center estimation remains feasible using the concentric arcs in the unsaturated region. Even so, estimation errors still occur due to non-ideal optical paths, noise, and objective misalignment in OBMs. The light source and IS of the OBM are inherently aligned, thus optical path alignment primarily involves the adjustment of the objective’s optical axis. Aligned optical path maximizes its aperture and transmitted optical power, as evidenced by the comparison of spot images in Figure 1. Hence, the estimation error of the spot center can be corrected by the position corresponding to the maximum the received intensity.

Imaging-feature-based RO identification and alignment schemes for OBMs encounter practical challenges: inadequate dynamic range of exposure levels and intensity of light source. To ensure robust RO identification and alignment across a broad dynamic range of exposure levels and light intensities, in this study, we delved into the spot features of OBMs at various magnifications and proposed a novel optical path alignment scheme. The scheme integrates the two aforementioned alignment guides, namely, spot features and received intensity, and achieves RO identification and alignment guidance for the optical path in OBMs using only an IS. Fundamentally, it simplifies the structure of the electric revolving nosepiece, bridges the gap in image-feature-based optical path alignment for OBMs, and provides robust support for the intelligent upgrade of biological microscopes. The specific contributions can be summarized as follows:

- Our paper introduces a novel and comprehensive scheme that simplifies the structure of optical path alignment in OBMs by utilizing only the built-in IS for both RO identification and alignment, bypassing the need for marker detection.
- We construct a model to simulate the movement of the light spot as the objective rotates near the optical axis and clarify the impact of objective length on the probability distribution of the spot edge position on the IS as well as the influence of the objective magnification—specifically 4× and 10×—on the gradient of the spot edge.
- An objective identification scheme that boasts a broad dynamic range, accommodating varying light source intensities and exposure levels, is proposed. Concretely, an evaluation function for zooming in on the imaging difference between 4× and 10× objectives is devised by integrating two dimensions: the weighted global variance and the approximation of the specific proportion of the bright area.
- We devise a weight distribution scheme for the concentric arcs extracted from the spot to enhance the accuracy of center estimation. Additionally, we uncover a phenomenon: the variation in received optical energy by the IS tends to zero as the optical path alignment of the RO improves. Accordingly, an evaluation function that is predicated on the variation in received energy is designed to generate alignment evaluation curve with sharp peak. These two elements are tasked with managing the fine and rough adjustment stages for the RO alignment, respectively.

The organization of this paper is as follows: In Section 2, we model and analyze the impact of the magnification and length of the objectives on imaging and introduce a spot-feature-based identification method specifically for the 4× objective. Section 3 elaborates on the rough alignment process for the 4× objective, which includes steps such as arc extraction and weighted circle fitting. Additionally, this section details the fine alignment process. Section 4 presents the experimental configurations and results, along with the corresponding analyses and discussions. Finally, Section 5 offers the conclusions and future outlooks.

Notation: In this paper, $\left\lfloor *\right\rfloor$ and $\langle *\rangle$ signify the operations of rounding down and element-wise summation, respectively. • denotes the operation of the dot product. ${∥*∥}_{2}$ represents the Euclidean norm. ${\left(*\right)}^{T}$ is the transpose operation. $1_{n}$ is an n×1 column vector with all elements being 1. The mean value of a matrix is signified by $\bar{*}$.

## 2. Identification Scheme for Reference Objective

The conventional objectives employed in OBMs include 4×, 10×, 20×, 40×, and 100×. Among these, the 4× objective provides the widest FOV, which is most convenient for locating target regions and is best suited as the reference objective. In an air setting without obstruction from slides and using a condenser of low numerical aperture, objectives with magnifications of 40× and above tend to completely darken the images, making them easily excluded. Similarly, the idle jacks for objectives in the revolving nosepiece are generally filled with dust covers and do no interfere with the RO identification. Hence, the RO identification is fundamentally streamlined to the task of differentiating between 4× and 10× objectives.

### 2.1. The Movement Characteristics of a Light Spot on an IS

**Figure 2:**
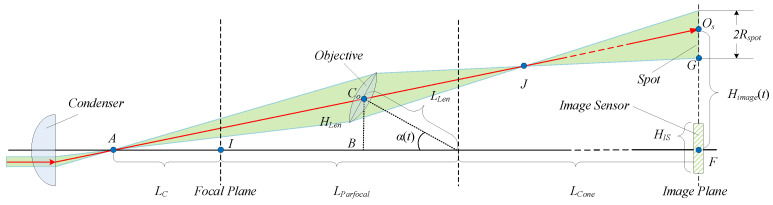
Movement model of the light spot as the objective rotates near the optical axis.

In this study, the misaligned optical path of OBM is simplified to a naïve model depicted in Figure 2. In this model, critical illumination is employed, and only the ray passing through the objective center is considered, regardless of the impact of the aperture.

$L_{Len}$, $L_{Parfoal}$, $L_{Cone}$, and $L_{C}$ represent the objective lengths, the parfocal distance, the lens cone, and the distance between the condenser and the focal plane, respectively. The illuminating beam is converged at point *A* by the condenser. AF and $FO_{s}$ represent the main optical axis and the image plane. α(t) is the deviation angle of the objective from the main optical axis at time *t*. The ray transmitting out of point *A* and passing through the optical center $C_{o}$ of the objective forms a spot center $O_{s}$ on the image plane. $H_{image}\left(t\right)$ represents the vertical range from point $O_{s}$ to the main optical axis. $H_{IS}$ denotes the width of the IS. When the revolving nosepiece rotates at angular velocity ω, point $O_{s}$ moves at velocity ϵ on the image plane. ϵ approximates to the velocity of the edge point closest to the main optical axis, denoted by point *G*, under the premise of extremely small spot deformation. This premise is true when the spot moves to the vicinity of the optical axis. Accordingly, $\Delta ABC∼\Delta AFO_{s}$, which means AB:AF = BC:$FO_{s}$; thus,

(1) $$\frac{L_{C}+L_{Parfoal}-L_{Len}\cos[\alpha \left(t\right)]}{L_{C}+L_{Parfoal}+L_{Cone}}=\frac{L_{Len}\sin[\alpha \left(t\right)]}{H_{image}\left(t\right)}.$$

ϵ is given by

(2) $$\begin{array}{c} \epsilon =\frac{dH_{image}\left(t\right)}{dt}=\frac{\left(L_{C}+L_{Parfoal}+L_{Cone}\right)L_{Len}{\left[\alpha \left(t\right)\right]}^{\prime }\left[\left(L_{C}+L_{Parfoal}\right)\cos\left[\alpha \left(t\right)\right]-L_{Len}\right]}{{\left(L_{C}+L_{Parfoal}-L_{Len}\cos\left[\alpha \left(t\right)\right]\right)}^{2}}. \end{array}$$

$\alpha \left(t\right)=\int_{0}^{t}\omega dx$, and it is relatively small as the objective center approaches the main optical axis; hence, cosωt≈1, and Equation (2) is simplified as

(3) $$\epsilon \approx \frac{\left(L_{C}+L_{Parfoal}+L_{Cone}\right)L_{Len}\omega }{L_{C}+L_{Parfoal}-L_{Len}}.$$

When point *F* traverses horizontally across the interval $\left[-\frac{H_{IS}}{2},\frac{H_{IS}}{2}\right]$ at ϵ, *N* transitional frames that contain point *G* are captured at a frame rate of $f_{ps}$, as formulated by

(4) $$N=\frac{H_{IS}f_{ps}}{\epsilon }=\frac{H_{IS}f_{ps}\left(L_{C}+L_{Parfoal}-L_{Len}\right)}{\left(L_{C}+L_{Parfoal}+L_{Cone}\right)L_{Len}\omega }.$$

*N* increases as $L_{Len}$ decreases. Since 4× and 10× objectives are conspicuously distinctive in length, the corresponding numbers of captured frames that contain the point *G*, signified by $N_{4\times }$ and $N_{10\times }$, are also distinct.

**Figure 3:**
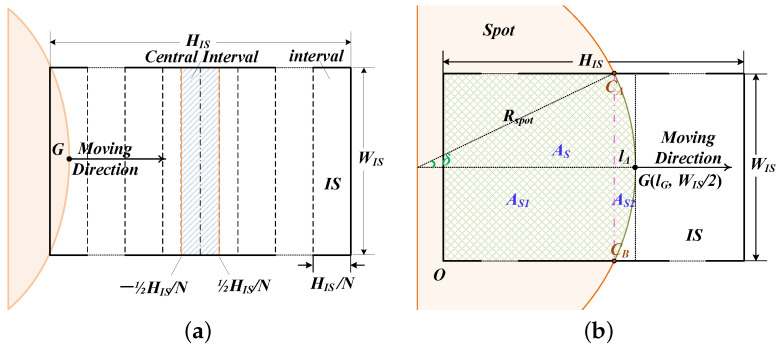
Spot movement on IS. (**a**) IS partition. (**b**) The coverage of IS by the light spot.

Note that the regions where the point *G* appears typically exhibit a high intensity gradient, and this gradient characteristic is the core criterion for RO identification. Figure 3a illustrates the movement of spots on an N-partition IS. The point *G* appears inevitably once in an arbitrary interval of $\left[l-\frac{H_{IS}}{2N},l+\frac{H_{IS}}{2N}\right]$, with *l* being a random horizontal position. Specifically, l=0 corresponds to the central interval of $\left[-\frac{H_{IS}}{2N},\frac{H_{IS}}{2N}\right]$. Hence, the probability density of the point *G* occurring at *l* is $\frac{1}{H_{IS}/N}$.

As for a M× objective, the probability that the point *G* appears in the detection interval of [−ξ/2,ξ/2] is $P_{M\times }=\frac{\xi N_{M\times }}{H_{IS}}$. Obviously, $P_{4\times }$ > $P_{10\times }$. When the detection interval is reduced below $\left[-\frac{H_{IS}}{2N_{4\times }},\frac{H_{IS}}{2N_{4\times }}\right]$, both $P_{4\times }$ and $P_{10\times }$ are less than 1. This implies the central interval may fail to capture the point *G* associated with the 4× objective or only the one corresponding to the 10× objective. Consequently, this may lead to the edge characteristics of the 4× objective being less conspicuous than those of the 10× objective, potentially resulting in misjudgments during RO identification. To mitigate this issue, the optimal detection interval should be set to $\left[-\frac{H_{IS}}{2N_{4\times }},\frac{H_{IS}}{2N_{4\times }}\right]$.

Owing to the random orientation of the IS on the imaging plane, the trajectory of the spot as it traverses the IS is inherently unpredictable. Ideally, the trajectories of all light spots tend to converge in the central region. Consequently, allocating higher weights to the central region during RO identification strengthens the contrast in gradient statistics and diminishes the risk of misjudgment.

When the spot traverses horizontally across the IS, the corresponding coverage effect on the IS is as illustrated in Figure 3b, where $l_{\Delta }$ is the sagitta. The intersection area $A_{S}$ between the spot and the IS is segmented into two separate parts, $A_{S1}$ and $A_{S2}$, by the chord $C_{A}C_{B}$. These areas can be determined by the geometric relationship between the circular spot and the rectangular IS. Accordingly, the proportion of the bright region when point *G* locates at $l_{G}$, denoted by $\gamma (l_{G})$, is formulated as

(5) $$\left\{\begin{array}{c} \gamma \left(l_{G}\right)=\frac{\overset{A_{S1}}{\overset{︷}{\left(l_{G}-l_{\Delta }\right)W_{IS}}}+\overset{A_{S2}}{\overset{︷}{\delta R_{spot}^{2}-\frac{W_{IS}}{2}\sqrt{R_{spot}^{2}-{\left(\frac{W_{IS}}{2}\right)}^{2}}}}}{W_{IS}H_{IS}}\\ l_{\Delta }=R_{spot}-\sqrt{R_{spot}^{2}-{\left(\frac{W_{IS}}{2}\right)}^{2}} \end{array}\right.\begin{array}{cc} , & l_{G}\in \left[l_{\Delta },H_{IS}-l_{\Delta }\right] \end{array},$$

where $R_{spot}$ is the spot radius and is given in the next section.

Essentially, $A_{S}$ represents a bright region on the IS with its area being contingent upon the position of the edge point *G*. In this context, the bright region is defined as the region inside which the gray values of pixels are non-zero. Accordingly, the area of the dark region is denoted by ${\bar{A}}_{S}=W_{IS}H_{IS}-A_{S}$. Given that $N_{4\times }>N_{10\times }$, the demarcation between ${\bar{A}}_{S}$ and $A_{S}$ by the spot edge formed by the 4× objective is more refined. Thus, in this study, we employed the proximity of $A_{S}$ to a particular value as a crucial criterion for further differentiating between 4× and 10× objectives.

### 2.2. The Edge Feature of a Light Spot

**Figure 4:**
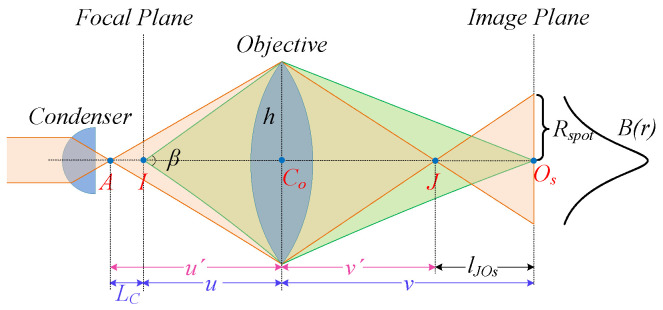
Optical path of a single-lens model.

The OBM objectives can be simplified to a single-lens model, as depicted in Figure 4. Here, point *I* signifies a point source located on the focal plane. The rays emanating from point *I* converges crisply at point $O_{s}$ on the image plane. Point *A* denotes the convergent point of the rays originating from the condenser, which can be approximated as a point source. Its convergent counterpart in the image space is designated as point *J*.

Point *A* is located outside the focal plane, creating a dispersive spot on the image plane. Let $l_{JO_{s}}$ denote the distance between point *J* and point $O_{s}$. According to the lens imaging law, $l_{JO_{s}}$ is expressed as

(6) $$\;l_{JO_{s}}=v-v^{\prime }=v-\frac{1}{\frac{1}{u}+\frac{1}{v}-\frac{1}{u^{\prime }}}.$$

In this context, *u* and $u^{\prime }$ represent the object distances for point *I* and point *A*, respectively. *v* and $v^{\prime }$ correspond to their respective image distances, where *u*, *v* and $L_{C}$ are prior values. In an air medium, the aperture angle β is given by β=arcsin(NA), where NA represents the known numerical aperture of the objective. The equivalent lens radius, denoted by $R_{Lens}$, is given by $R_{Lens}=u\tan\left(\beta /2\right)$. Thus, the radius of spot is $R_{spot}=\frac{l_{JK}R_{Lens}}{v^{\prime }}$.

The intensity distribution inside the spot can be described by $B\left(r\right)=\frac{1}{2\pi {\sigma_{B}}^{2}}\exp\left(-\frac{r^{2}}{2{\sigma_{B}}^{2}}\right)$, where $r=\sqrt{x^{2}+y^{2}}$, *x*,*y* are the two-dimensionL coordinates on the image plane. $\sigma_{B}$ is the standard deviation of the Gaussian function. The intensity gradient of the spot edge is given by

(7) $$\;B^{\prime }\left(R_{spot}\right)=\left(-\frac{R_{spot}}{{\sigma_{B}}^{2}}\right)\frac{1}{2\pi \sigma^{2}}\exp\left(-\frac{R_{spot}^{2}}{2{\sigma_{B}}^{2}}\right),$$

with $R_{spot}$ being proportional to $\sigma_{B}$, i.e., $\sigma_{B}=qR_{spot}$ [31]. Therefore, Equation (7) is reformulated as

(8) $$B^{\prime }\left(R_{spot}\right)=-\frac{1}{2\pi q^{4}R_{spot}^{3}}\exp\left[-\frac{1}{2q^{2}}\right].$$

Typically, $R_{spot\_10\times }\approx 2.5R_{spot\_4\times }$, where $R_{spot\_M\times }$ denotes the spot radius for the M× objective. Accordingly, $\frac{{B^{\prime }}_{4\times }\left(R_{spot\_4\times }\right)}{{B^{\prime }}_{10\times }\left(R_{spot\_10\times }\right)}=15.625$. This implies that the intensity gradient at the spot edge formed by 4× objective is up to 15.6 times larger than that formed by 10× objective. Actually, the gradient variation between the two objectives is more pronounced due to the diffraction effects along the optical path. Thus, the remarkable distinction in the edge gradient may serve as a viable criterion for RO identification.

### 2.3. RO Identification Scheme

The RO identification scheme is meticulously crafted to consider two pivotal dimensions: the gradient statistics of spot images and the granularity of bright and dark region segmentation. Given that each interval on the IS is theoretically equivalent, the efficacy of RO identification remains consistent regardless of which interval is chosen for the analysis of edge features and the ratio of bright to dark pixels. Accordingly, a $H_{im}\times W_{im}$ spot image with a serial number *k* is divided into $N_{4\times }^{\prime }\times M_{4\times }^{\prime }$ sub-blocks, where $N_{4\times }^{\prime }=\left\lfloor N_{4\times }\right\rfloor$, $M_{4\times }^{\prime }=\left\lfloor M_{4\times }\right\rfloor$. The corresponding local variance matrix is denoted by $\Psi_{k}$, with the *i*,*j*-th element being signified by $\sigma_{i,j}$. Each sub-block is assigned a weight $\omega_{i,j}$ from a predefined weight matrix $W_{G}$. Note that a certain sub-block rich in edges is assigned a greater weight. Then, the overall variance is determined for the gradient statistics of the spot images. To align with the weight matrix, an indicator, denoted as *g*, is incorporated into the evaluation function for RO identification, as summarized by

(9) $$\;\sigma_{p\_k}^{2}=\frac{{∥W_{G}\cdot \Psi_{k}-\bar{\langle W_{G}\cdot \Psi_{k}\rangle }1_{{N^{\prime }}_{4\times }}1_{{M^{\prime }}_{4\times }}^{T}∥}_{2}}{g_{k}},g_{k}=\left|\frac{S_{C\_k}-T_{\gamma }}{T\gamma }\right|,$$

where $T_{\gamma }=\gamma_{4\times }\left(l_{G}^{\prime }\right)H_{im}W_{im}$. $T_{\gamma }$ represents the number of pixels in the bright region when the point *G* is located at $\left(l_{G}^{\prime },W_{IS}/2\right)$. $T_{\gamma }$ is associated with the weight matrix for the 4× objective. $S_{C\_k}$ denotes the statistics of bright pixels in the *k*-th spot image. As for the 4× objective, when the overall variance inof the *k*-th spot image reaches its extremum, the corresponding $S_{C\_k}$ closely matches $T_{\gamma }$, and, concurrently, the $\sigma_{p\_k}^{2}$ is conspicuously high and maximized. Conversely, for objectives with a magnification of 10× or greater, the overall variances are inherently small, and the numbers of bright pixels struggle to approximate $T_{\gamma }$. Consequently, the evaluation value remains consistently low.

Despite the fact that the motion of the light spots is not necessarily parallel to the horizontal axis of the IS, all trajectories of the spots ultimately meet at the central area of the IS. This fact makes the central area an ideal candidate for intensive detection. To enhance the manageability of the weight assignments and focus gradient statistics on the central area of the IS, we employ a normalized two-dimensional Gaussian function with a variance $\sigma_{H}^{2}$ to serve as the weight matrix for the sub-blocks. Accordingly, $l_{G}^{\prime }$ is set to $H_{IS}/2$. The complete process for RO identification is presented in Algorithm 1. Note that $T_{\sigma_{p\_k}^{2}}$ is a threshold for $\sigma_{p\_k}^{2}$, $I_{m\_k}$$\left(i,j\right)$ is the i,j-th sub-block in *k*-th spot image.
**Algorithm 1** RO identification scheme**Input: **$I_{m\_k}$, $H_{im}$, $W_{im}$, $N_{4\times }^{\prime }$, $\sigma_{H}^{2}$;**Output: **$O_{type}$;  1: $W_{S}=W_{im}/M_{4\times }^{\prime }$; $H_{S}=H_{im}/N_{4\times }^{\prime }$; $O_{type}=0$;  2: $w_{i,j}^{\prime }=\frac{1}{2\pi \sigma_{H}^{2}}\exp\left(\frac{{\left(i-\frac{1+{M^{\prime }}_{4\times }}{2}\right)}^{2}+{\left(j-\frac{1+{N^{\prime }}_{4\times }}{2}\right)}^{2}}{2\sigma_{H}^{2}}\right)$; $w_{i,j}=\frac{w_{i,j}^{\prime }}{\langle w_{i,j}^{\prime }\rangle }$;  3: **while** $\sigma_{p\_k}^{2}$ > $T_{\sigma_{p}^{2}}$ **do**  4:      *k*++;  5:      $O_{type}=0$;  6:      $\sigma_{i,j\_k}^{2}={∥I_{m\_k}\left(i,j\right)-\bar{I_{m\_k}\left(i,j\right)}1_{W_{S}}1_{H_{S}}^{T}∥}_{2}$;  7:      $g_{k}={\left.\left|\frac{{∥I_{m\_k}∥}_{0}-T_{\gamma }}{T_{\gamma }}\right|\right|}_{{l^{\prime }}_{G}=H_{IS}/2}$;  8:      $\sigma_{p\_k}^{2}=\frac{{∥W_{G}\cdot \Psi_{k}-\bar{\langle W_{G}\cdot \Psi_{k}\rangle }1_{{N^{\prime }}_{4\times }}1_{{M^{\prime }}_{4\times }}^{T}∥}_{2}}{g_{k}}$;  9: **end while**10: $O_{type}=1$;11: **return** $O_{type}$.

## 3. Alignment Scheme for Reference Objective

RO alignment is accomplished through a synergistic approach that integrates spot center positioning with received light intensity, delineating the process into two distinct stages: rough and fine alignments. During the rough alignment stage, the spot center is estimated by extracting concentric arcs and performing weighted circle fitting. In the fine alignment stage, a sharp curve for alignment evaluation is constructed to highlight the optimal alignment position when the received optical power is unsaturated. Upon reaching saturation of the received optical power, the optimal alignment position is inferred from paired critical saturation positions.

### 3.1. Rough Alignment for RO: Weighted Circle Fitting

**Figure 5:**
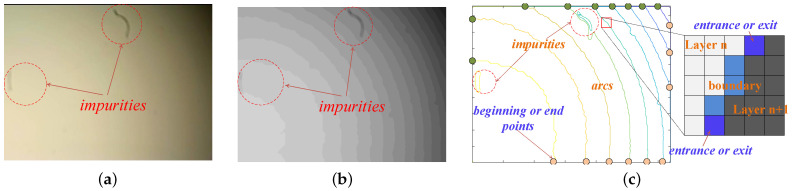
Arc extraction and impurity interference. (**a**) Spot image with impurities. (**b**) Grayscale stratification. (**c**) Arc extraction.

Intensity distribution inside a spot obeys the two-dimensional Gaussian distribution; thus, a group of concentric arcs can be extracted from the spot in accordance with the gray levels. To enhance the clarity of layering within the spot image and to facilitate precise arc extraction, grayscale quantization, median filtering, and edge detection are employed in image preprocessing, as illustrated in Figure 5a,b.

Arc extraction is susceptible to interference from imaging impurities, as illustrated by the lens impurities in Figure 5b. These impurities form short and isolated curves that distort the arcs. Each arc typically has paired endpoints, such as the orange and green points in Figure 5c, which are ideally positioned at the edges of the image. However, impurity-induced curves seldom exhibit this characteristic. The arc extraction process, which initiates at a random endpoint and proceeds with an eight-neighbor search until it reaches another endpoint, is an effective method for filtering out the curves formed by impurities. Moreover, certain impurity curves that have paired endpoints can still be distinguished from genuine arcs by their shorter lengths. Typically, arcs act as layer boundaries without any branching, leading to a matrix structure that features a single entry point and a single exit point spanning across two layers. This is exemplified by the 5 × 5 matrix in Figure 5c.

**Figure 6:**
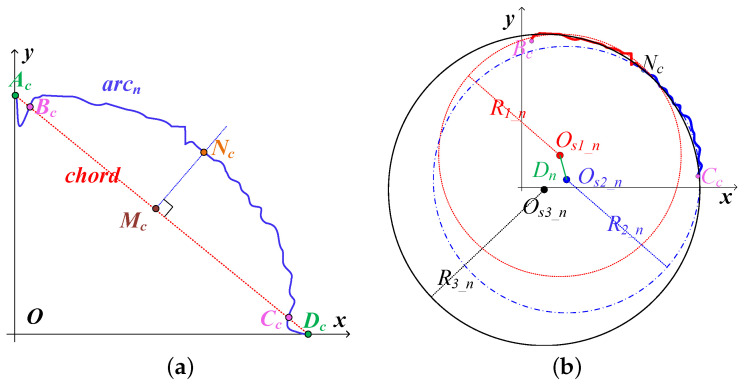
Weight design of concentric arcs. (**a**) Arc cutting. (**b**) Arc symmetry analysis.

Some of the extracted arcs inherently undergo distortion due to a non-ideal optical path. Hence, we assess the distortion severity by the symmetry of the arcs and accordingly allocate weights to them that are proportional to their distortion levels. To enhance the precision of the endpoints, we identify the pair of intersections closest to the chord’s midpoint among the points where the chord intersects the arc. These identified intersections are designated as the new endpoints, and the arc is subsequently adjusted, as illustrated in Figure 6a. It should be noted that points $A_{c}$ and $B_{c}$ are the initial endpoints, while points $C_{c}$ and $D_{c}$ are the updated endpoints. Point $M_{c}$ denotes the midpoint of chord $B_{c}C_{c}$, and the perpendicular bisector of $B_{c}C_{c}$ intersects the arc at point $N_{c}$.

Least squares circle fitting is applied to $\overset{⌢}{B_{c}N_{c}}$, $\overset{⌢}{N_{c}C_{c}}$, and $\overset{⌢}{B_{c}C_{c}}$, with the resulting center positions denoted as $O_{s1\_n}$, $O_{s2\_n}$, and $O_{s3\_n}\left(X_{O_{s3\_n}},Y_{O_{s3\_n}}\right)$, respectively. Correspondingly, the radii of these circles are $R_{1\_n}$, $R_{2\_n}$, and $R_{3\_n}$. The Euclidean distance between $O_{s1\_n}$ and $O_{s2\_n}$ is represented by $D_{n}$. The greater the disparity between $R_{1\_n}$ and $R_{2\_n}$ and the larger the distance between them, the less symmetrical the circular arc becomes. With this in mind, the weight assignment for the circular arc is formulated as

(10) $$W_{n}=\frac{\Omega_{n}}{\sum_{n=1}^{U}\Omega_{n}},\Omega_{n}=\frac{\min\left(R_{1\_n},R_{2\_n}\right)}{D_{n}\min\left(R_{1\_n},R_{2\_n}\right)}.$$

The weighted center position of the *k*-th spot image, denoted by $O_{sk}\left(X_{O_{sk}},Y_{O_{sk}}\right)$, is described as $O_{sk}=$$W_{n}$${\left[O_{s3\_1},\cdots ,O_{s3\_U}\right]}^{T}$. Adjust the revolving nosepiece to direct the spot’s center from $\left(X_{O_{sk}},Y_{O_{sk}}\right)$ to $\left(H_{im}/2,W_{im}/2\right)$, thereby achieving the rough alignment for the RO.

### 3.2. Fine Alignment for RO: Received Intensity Variation for Alignment Evaluation

As the spot center approaches the position of optimal alignment, the variation in the received optical power slows or even ceases due to saturation, resulting in an evaluation curve with a flattened peak. This presents a challenge for aligning the optical path. The key to achieving precise alignment lies in accurately identifying and locating the peak position, both in unsaturated and saturated conditions.

The received optical power is reflected in the image energy. The image energy can be characterized by the integral of a two-dimensional Gaussian function, parameterized by $\sigma_{F}$, over a rectangular area, as represented by $E(X_{O},Y_{O})$ in ReceivedPowerIntegral. The horizontal orientation of the IS may not coincide with the movement direction of the spot, as exemplified by the actual spot images in Figure 5 and Figure 6, where the spot center is noticeably off the horizontal midline of the IS. To reveal the correlation between the energy statistics of spot images and the alignment degree of the optical path, $E(X_{O},Y_{O})$ is decomposed into independent integrals along the horizontal and vertical directions, denoted by $E_{H}\left(X_{O}\right)$ and $E_{V}\left(Y_{O}\right)$, as detailed in Equation (11). The decomposition primarily hinges on the property of the dimensional independence characteristic of rectangular integration regions.

(11) $$E\left(X_{O},Y_{O}\right)=\int_{0}^{H_{im}}{\int_{0}^{W_{im}}\left(\frac{1}{\sqrt{2\pi }\sigma_{F}}e^{\frac{{\left(x-X_{O}\right)}^{2}+{\left(y-Y_{O}\right)}^{2}}{2\sigma_{F}^{2}}}\right)}^{2}dxdy=\underset{E_{H}\left(X_{O}\right)}{\underset{︸}{\left(\frac{1}{\sqrt{2\pi }\sigma_{F}}\int_{0}^{H_{im}}e^{\frac{{\left(x-X_{O}\right)}^{2}}{\sigma_{F}^{2}}}dx\right)}}\underset{E_{V}\left(Y_{O}\right)}{\underset{︸}{\left(\frac{1}{\sqrt{2\pi }\sigma_{F}}\int_{0}^{W_{im}}e^{\frac{{\left(y-Y_{O}\right)}^{2}}{\sigma_{F}^{2}}}dy\right)}}.$$

**Figure 7:**
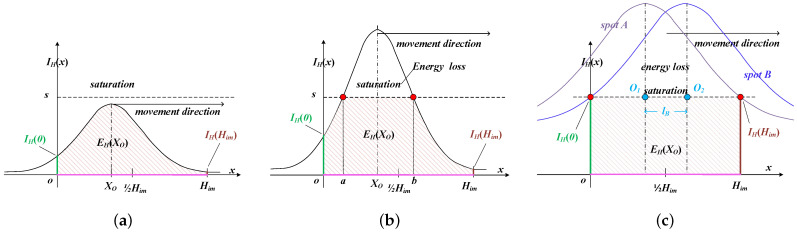
Received optical power under various states. (**a**) Unsaturated state. (**b**) Partially saturated state. (**c**) Completely saturated state.

In this paper, we concentrate on analyzing the energy variation within the horizontal dimension in spot images, with a parallel approach applied to the vertical dimension. With the increase in light intensity or exposure level, the IS gradually transitions from an unsaturated state into a partially saturated state, as depicted in Figure 7a,b. Here, *a* and *b* represent the horizontal boundaries of the saturated area within $\left[0,\;H_{im}\right]$. The shaded area represents the captured energy of the spot image when observed horizontally, with $I_{H}\left(0\right)$ and $I_{H}\left(H_{im}\right)$ indicating the captured energy at the respective horizontal boundaries of the spot image.

As the spot center moves directly toward the IS center, the captured energy changes. Notably, the variations in captured energy in both the unsaturated and partially saturated states are identically formulated by

(12) $$E_{H}^{\prime }\left(X_{O}\right)=\frac{1}{\sqrt{2\pi }\sigma_{F}}\int_{0}^{H_{im}}d\left[\frac{e^{\frac{{\left(x-X_{O}\right)}^{2}}{\sigma_{F}^{2}}}}{dX_{O}}\right]dx=I_{H}\left(0\right)-I_{H}\left(H_{im}\right),$$

where $I_{H}\left(x\right)=\frac{1}{\sqrt{2\pi }\sigma_{F}}\exp\left[{\left(x-X_{o}\right)}^{2}/\sigma_{F}^{2}\right]$. Indeed, regardless of the saturation state, the instantaneous change in energy is essentially the difference between the energy gain at $I_{H}\left(0\right)$ and the energy loss at $I_{H}\left(H_{im}\right)$ as the spot traverses the pattern depicted in Figure 7. As $X_{O}$ approaches $\frac{1}{2}H_{im}$, the energy changes at both ends tend to converge due to the symmetry of the Gaussian model, leading to $E_{H}^{\prime }\left(X_{O}\right)$ decreasing to 0. The same applies to $E_{V}^{\prime }\left(Y_{O}\right)$. In this study, this property was utilized to sharpen the evaluation curve of optical path alignment. The evaluation function for alignment degree is formulated as

(13) $$S_{k}=\left\{\begin{array}{cc} \frac{E_{k}}{E_{k}-E_{k-1}} & ,\\ 0 & , \end{array}\right.\begin{array}{cc} & \begin{array}{c} E_{k}⩾E_{k-1}\\ E_{k}<E_{k-1} \end{array} \end{array},$$

where $E_{k}={∥I_{m\_k}∥}_{2}^{2}$ is the energy of the spot image captured in the *k*-th move of the objective. The peak position of $S_{k}$ instructs the aligned state.

High exposure levels and strong light intensities saturate the IS, resulting in no variation in the captured energy as the revolving nosepiece rotates, as illustrated in Figure 7. In this state, the IS was already saturated prior to the full alignment of the objective, giving rise to peak clipping for the $E_{H}\left(X_{O}\right)$ curve. This premature saturation introduces indistinctness between the aligned and misaligned states. A reasonable reduction in exposure level or light intensity can intuitively suppress the peak clipping effect. However, there is no universal criterion for what constitutes ‘reasonable’ reductions. The exposure level and light intensity lack versatility for all microscopic systems, and suitable values for these two parameters require prior information about the photosensitivity of specific microscopic systems, which depends on the characteristics of the optical path, light source, and IS.

To simplify the requirements for prior system parameters, once all pixels are saturated, we turn to identify the paired critically saturated positions rather than the peak position. In Figure 7, $I_{H}\left(H_{0}\right)$ and $I_{H}\left(H_{im}\right)$ represent the paired critically saturated positions, with $O_{s1}$ and $O_{s2}$ marking the centers of spotA and spotB, respectively. Owing to the symmetry inherent in the Gaussian function, the midpoint between $O_{s1}$ and $O_{s2}$ corresponds precisely to the alignment position of the optical path. Therefore, the optical path alignment is accomplished by shifting the spot center back by a distance of $\frac{1}{2}l_{B}$ from the critical saturation position. Thus, in the fine alignment stage, different evaluation schemes are chosen based on the situations of the completely saturated state and incompletely saturated state.

## 4. Experimental Analysis

**Figure 8:**
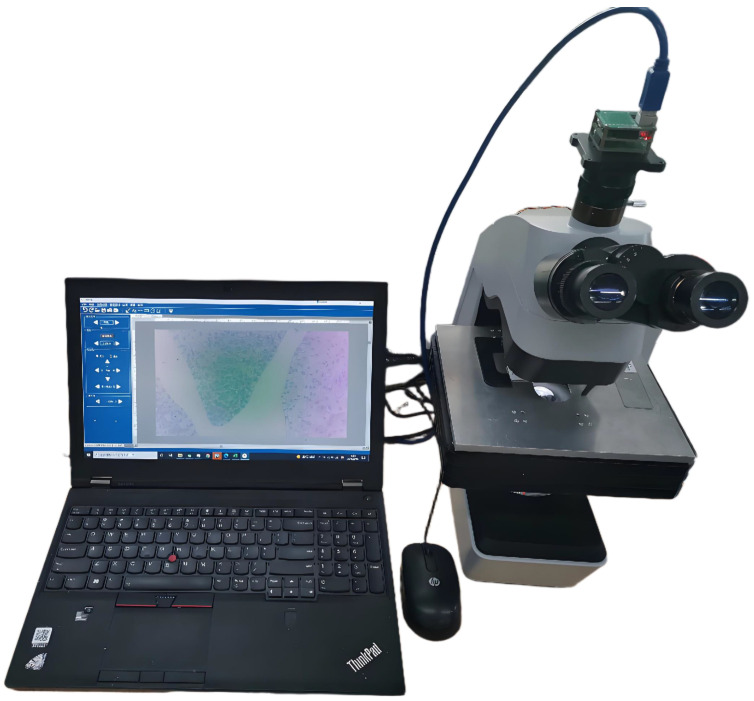
Microscopic workstation.

A microscopic workstation, jointly developed by Wuzhou University and OKA Optical Instruments Manufacturing in Wuzhou, China, served as the experimental platform to evaluate the performance of the proposed method, as depicted in Figure 8. The specific experimental parameters are detailed in Table 1. The experimental image sequences were captured using AMCap software of version 9.23 on the Windows 7 platform, with the exposure levels being divided into 8 levels and marked from −4 to −11 as designated by AMCap.

### 4.1. Identification of 4× and 10× Objectives

**Figure 9:**
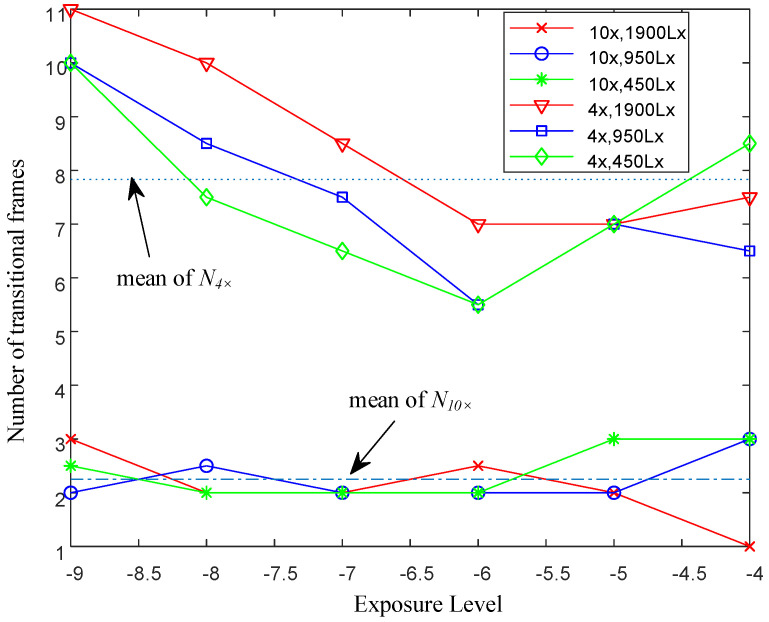
Number of transitional frames captured by 4× and 10× objectives at different light intensities and exposure levels.

Under the experimental conditions specified in Table 1, the theoretical values of $N_{4\times }$ and $N_{10\times }$ derived from Equation (4) are 2.4 and 7.2, respectively. Figure 9 illustrates the number of transitional frames captured by the 10× and 4× objectives under varying light intensities and exposure levels.

**Figure 10:**
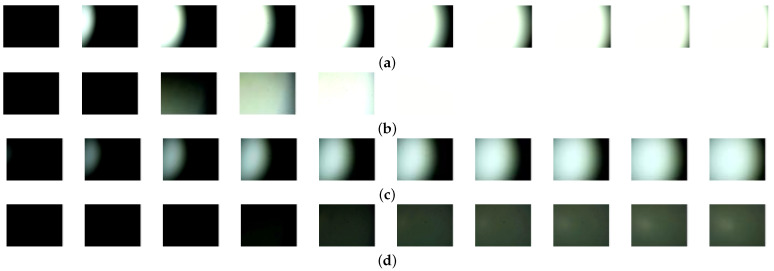
Transitional image sequences captured under different light intensities and exposure levels. (**a**) Transitional sequence captured by 4× objective under 950 Lx and exposure level of −6. (**b**) Transitional sequence captured by 10× objective under 950 Lx and exposure level of −6. (**c**) Transitional sequence captured by 4× objective under 1900 Lx and exposure level of −10. (**d**) Transitional sequence captured by 10× objective under 1900 Lx and exposure level of −10.

The average number of the actual transitional frames captured by the two objectives under the aforementioned conditions were 2.26 and 7.8, respectively. The differences between experimental and theoretical values were minimal, not exceeding one frame. For the 10× objective, only two to three transitional frames are captured, suggesting a low likelihood of the spot edge appearing centrally in the spot image. In contrast, the 4× objective captured seven to eight transitional frames, increasing the probability of the spot edge being closer to the image center. The transitional image sequences, captured at 950 Lx with an exposure level of −6 and at 1900 Lx with an exposure level of −10, are depicted in Figure 10.

Figure 10 reveals distinct imaging traits between the 4× and 10× objectives as they move around the main optical axis. The 4× objective consistently yielded sharper spot edges regardless of light intensities and exposure settings. In contrast, the 10× objective exhibited faster coverage of the IS.

**Figure 11:**
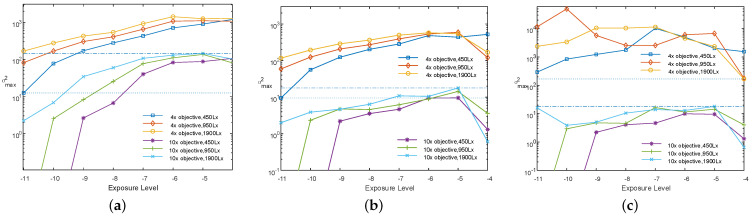
Exposure level–$\sigma_{max}^{2}$ curves corresponding to 10× and 4× objectives at different light intensities and exposure levels. (**a**) Neither the weight matrix nor parameter *g* is involved in the computation of $\sigma_{p}^{2}$. (**b**) Only the weight matrix is involved in the computation of $\sigma_{p}^{2}$. (**c**) Both the weight matrix and parameter *g* are involved in the computation of $\sigma_{p}^{2}$.

The imaging difference between these two objectives is quantitatively characterized by the maximum of $\sigma_{p}^{2}$, denoted by $\sigma_{max}^{2}$. The exposure level–$\sigma_{max}^{2}$ curves for light intensities of 450 Lx, 950 Lx, and 1900 Lx are presented in Figure 11a–c. In the context of these figures, the dash-dotted lines represent the minima of exposure level–$\sigma_{max}^{2}$ curves corresponding to the 4× objective, whereas the dotted lines signify the maxima of the exposure level–$\sigma_{max}^{2}$ curves corresponding to the 10× objective.

The $\sigma_{max}^{2}$ corresponding to the 4× objective are generally much larger than those corresponding to the 10× objective under identical conditions, whereas this may not hold true under varying conditions. Referring to the two sets of curves in Figure 11, when neither the weight matrix nor the parameter *g* is involved in the computation of $\sigma_{p}^{2}$, the maxima of the $\sigma_{max}^{2}$ curves for the 10× objective are considerably larger than the minima of the $\sigma_{max}^{2}$ curves for the 4× objective. Consequently, there is no uniform thresholds that can be applied across all light intensities and exposure levels to distinguish between the two objectives. Though the involvement of a weight matrix in the computation of $\sigma_{p}^{2}$ broadens the gap in the range between the two sets of curves, as illustrated in Figure 11b, there are still areas of overlap. When parameter *g* is included in the computation of $\sigma_{p}^{2}$, the ranges of the two sets of curves become completely distinct, with a gap exceeding $10^{2}$, as demonstrated in Figure 11c. This provides a sufficient margin for the selection of a uniform threshold for objective identification. Exposure levels beyond the range of [−10, −6] result in images that are either too dark or too bright, which is not conducive to the subsequent fine alignment stage. Given that light intensities and exposure levels can be controlled, the valid exposure level is preset within the range of [−10, −6].

### 4.2. Rough Alignment for 4× Objective

**Figure 12:**
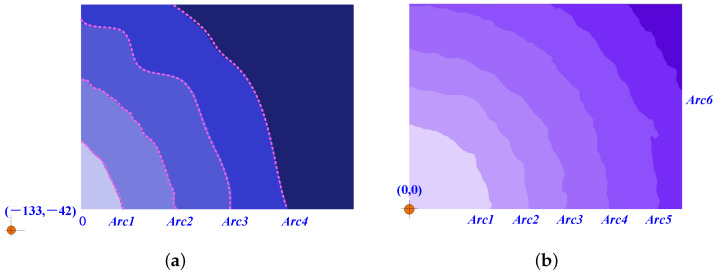
Light spot with several concentric arcs. (**a**) Spot center is located outside the IS. (**b**) Spot center is located at the corner of the IS.

The accuracy of rough alignment is largely determined by the precision of circle fitting through concentric arcs. Since actual spots do not have a reference center, we employed two sets of drawn concentric arcs, as shown in Figure 12c, to assess the accuracy of our proposed weighted circle fitting method. Each arc was intentionally subjected to varying degrees of random perturbations. The central coordinates for the arcs in Figure 12a,b are designated as (−133, −42) and (0, 0), respectively. The fitted central positions and the weighted central positions for each arc are detailed in Table 2 and Table 3.

The upper segment of Arc3 in Figure 12a exhibits significant distortion, with its fitting center markedly deviating from the actual center. This results in a relatively low weight factor being assigned. In contrast, Arc2 demonstrates the most pronounced symmetry and is therefore allocated the highest weight factor. The weighted fitting error is minimal, falling below two pixels, indicating a high degree of accuracy in the determination of the fitting center. Similarly, in Figure 12b, Arc6 and Arc1 are assigned the highest and lowest weight factors, respectively. The average distortion of the arcs in Figure 12b is more pronounced than in Figure 12a, leading to a relatively larger error. However, when compared to the spot radius, which can extend to several hundred pixels—approximately 667 pixels in Figure 12a and 996 pixels in Figure 12b—the center estimation error of a few dozen pixels is still within acceptable limits.

**Figure 13:**
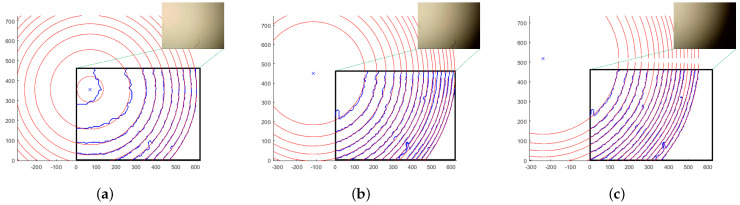
Weighted central fitting for actual light spot. (**a**–**c**) Weighted circle fitting effect under different spot positions.

Regarding the actual light spot, the arcs may not be perfectly concentric because the main optical axis is not entirely perpendicular to the imaging plane. Figure 13 illustrates several arcs that were extracted in practice. In Figure 13a, the fitting central position of the light spot, marked with a blue ‘×’, is located outside the IS, whereas in Figure 13b,c, it is located inside the IS. The red dotted lines in each subfigure represent a set of arcs that are strictly concentric around their respective fitting central positions. These concentric arc sets generally align well with the corresponding actual arc sets, indicating that the weighted circle fitting method meets the accuracy requirements for the rough alignment process for RO.

### 4.3. Optical Path Alignment of 4× Objective

**Figure 14:**
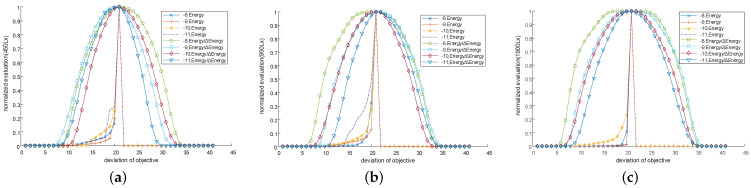
Normalized alignment evaluation curves under different light intensities and exposure levels. (**a**) Alignment evaluation curves at 450 Lx with exposure levels from −8 to −11. (**b**) Alignment evaluation curves at 950 Lx with exposure levels from −8 to −11. (**c**) Alignment evaluation curves at 1900 Lx with exposure levels from −8 to −11.

Under exposure levels ranging from −8 to −11 and light intensities of 450 Lx, 950 Lx, and 1900 Lx, the IS remains unsaturated or only partially saturated. Consequently, there is no peak clipping to distort the curve of the captured energy, as evidenced by the normalized curves in Figure 14.

Increasing either the exposure level or the light intensity tends to flatten the peaks of the curves representing the captured energy. This increased flatness, as observed in the three sets of curves in Figure 14, exacerbates the challenge of locating peaks. However, when the variation in captured energy is incorporated into the alignment evaluation as a denominator, the resulting evaluation curves become significantly sharper, as shown in Figure 14. This suggests that the evaluation method, which incorporates changes in energy, demonstrates greater robustness against variations in exposure levels and light intensities than the method that relies solely on captured energy. Given that evaluation curves with clipped peaks fail to indicate the aligned location under saturated conditions, we validated the effectiveness of the previously mentioned alignment estimation method, which is based on critical saturation positions, by inducing artificial saturation under a partially saturated state. This artificial saturation was simulated by extracting a central area of $W_{im}/2\times H_{im}/2$ from the $W_{im}\times H_{im}$ captured image and then applying peak clipping based on a predetermined graylevel threshold. This process is analogous to centrally cropping the IS to a quarter of its size and reducing the saturation threshold of the photoelectric sensor units. Table 4 lists the step errors of the aligned positions estimated using critical saturation positions for both unsaturated and partially saturated conditions.

The IS becomes saturated under the combined conditions of either 1900 Lx or 950 Lx with an exposure level of −7, resulting in an absence of a reference for optical alignment position. Additionally, artificial saturation is not achievable under 450 Lx with an exposure level of −11 due to the insufficient energy captured by the IS. In this case, the error is marked with an asterisk (*) in Table 4. Validating the performance of the alignment estimation method, which relies on critical saturation positions, was challenging under such conditions. The step errors under other combined conditions, as shown in Table 4 did not exceed 2, with the maximum average step error at 450 Lx being 1.125. Furthermore, the overall average step error across all the intensities was 0.875. These experimental results suggest a small bias in the estimation of the aligned position based on critical saturation positions and confirm the feasibility of the estimation method.

## 5. Conclusions

In this study, a comprehensive alignment strategy for the optical path of an OBM, encompassing the identification and alignment for the RO, specifically the 4× objective, was proposed. Within the RO identification phase, enhancing the gradient statistical weight for the central area of IS and incorporating a proportion factor associated with the bright region are measures that substantially differentiate the gradient disparities among spot images formed by a 4× objective and those formed by other objectives. In the optical path alignment phase, the intensity distribution of the spot is verified to approximate a two-dimensional Gaussian distribution. This feature facilitates the extraction of concentric arcs and the implementation of weighted circular fitting. Thus, the deviation of the optical path can be approximately determined. Within the context of a two-dimensional Gaussian distribution of light intensity, the proximity of the optical path to its optimal alignment position correlates with a diminished increase in image energy. This property is particularly advantageous as it can be leveraged to significantly sharpen the evaluation curve for optical path alignment. Exploiting the symmetry inherent in the two-dimensional Gaussian function, the optimal alignment position of the optical path can be accurately predicted by a pair of critical saturation positions. The comprehensive scheme adeptly bridges the gap in alignment guidance for the optical path of OBMs that rely on image processing technology, offering a novel dimension for the optical path alignment of OBMs. Furthermore, the test results from an actual microscope platform substantiate the efficacy of the proposed scheme. Since the obstruction of the specimen to the optical path was not considered in the current study, a key focus in a future study will be on achieving precise optical path alignment in the presence of specimen interference.

## Figures and Tables

**Table 1:** Experimental parameters.

Parameters	Values	Parameters	Values
Camera model	CMCMOS500	NA of 4× objective	0.1
Microscope model	OKA XSZ-702	NA of 10× objective	0.25
IS size	$1/2.5^{\prime \prime }$	Exposure level	[−4, −11]
Frame rate	30 $f_{ps}$	Light intensity (Lx)	450, 950, 1900
ω	0.45 rad/s	$\sigma_{H}$	2
$L_{Cone}$	160 mm	$H_{im}\times W_{im}$	640 × 480
$L_{parfoal}$	45 mm	Arc length constraint	≥150 pixels
$L_{Len(4\times )}$	13 mm	Grayscale for quantization	10
$L_{Len(10\times )}$	27 mm	Kernel of median filter	19 × 19
$L_{C}$	15 mm	Edge detection operator	Canny

**Table 2:** Weighted circle fitting for concentric arcs in Figure 13a.

	Arc1	Arc2	Arc3	Arc4	Evaluated Center	Actual Center	Error
$X_{O_{3\_n}}$	133.692	−137.956	−75.6366	−76.6798	−133.5386	−133	0.5386
$Y_{O_{3\_n}}$	−37.6416	−45.6159	−1.9927	−2.3814	−40.9078	−42	1.0922
$W_{n}$	0.321	0.6294	0.0096	0.0399			

**Table 3:** Weighted circle fitting for concentric arcs in Figure 13b.

	Arc1	Arc2	Arc3	Arc4	Arc5	Arc6	Evaluated Center	Actual Center	Error
$X_{O_{3\_n}}$	−7.4	27.2	5.28	33.34	125.6	406.5	5.02	0	5.02
$Y_{O_{3\_n}}$	−0.77	37.26	−7.07	48.9	64.8	274.97	10.4	0	10.4
$W_{n}$	0.656	0.0667	0.1061	0.0174	0.0038	0.15			

**Table 4:** Step errors of the aligned positions.

Intensity/Exposure	−7	−8	−9	−10	−11	Average
1900 Lx	saturated	0	0.5	0.5	1.5	0.625
950 Lx	saturated	0.5	1	1.5	0.5	0.875
450 Lx	0.5	2	0.5	1.5	*	1.125
average						0.875

## Data Availability

Data are contained within the article.
